# Supplementary material for: Prevalence and Determinants of Vitamin D Deficiency in 1825 Cape Town Primary Schoolchildren: A Cross-Sectional Study
Source: Nutrients. 2022 Mar 16;14(6):1263. doi: 10.3390/nu14061263 (PMC8952729; doi:10.3390/nu14061263)
Supplement: Supplementary file 1 [file nutrients-14-01263-s001.zip › nutrients-1604654-supplementary.pdf]

Table S1. Multivariate linear regression model of determinants of 25(OH)D deficiency (25[OH]D <50 nmol/L), excluding BMI-for-age Z-score variable.

Variable	B coefficient (95% Confidence Interval)	p-Value
**Sex**		
Male	0 (reference)	0.001
Female	-2.28 (-3.67 to -8.89)	
**Age at assent and screening (years)**	-1.47 (-2.05 to -0.90)	< 0.001
**Season screened**		
Jan-Mar	0 (reference)	< 0.001
Apr-Jun	-8.88 (-10.57 to -7.19)	
Jul-Sep	-22.05 (-24.08 to -20.02)	
Oct-Dec	-15.40 (-17.92 to -12.88)	
**Housing Type**		
Brick	0	0.15
Informal	+1.02 (+0.70 to +1.49)	

Table S2. Multivariate logistic regression model of determinants of 25(OH)D deficiency (25[OH]D <50 nmol/L), excluding BMI-for-age Z-score variable

Variable	Odds Ratio (95% Confidence Interval)	p-Value
**Sex**		
Male	1 (reference)	0.024
Female	1.57 (1.06 to 2.32)	
**Age at assent and screening (years)**	1.25 (1.05 to 1.49)	0.011
**Season screened**		
Jan-Mar	1 (reference)	< 0.001
Apr-Jun	2.62 (1.17 to 5.86)	
Jul-Sep	16.42 (7.99 to 33.74)	
Oct-Dec	7.95 (3.51 to 18.02)	
**Ethnicity**		
IsiXhosa	1 (reference)	0.64
Coloured	-	
Other	0.62 (0.072 to 5.27)	
**Margarine consumption**	0.85 (0.68 to 1.05)	0.13
